# Automatic Quality Control System and Adenoma Detection Rates During Routine Colonoscopy: A Randomized Clinical Trial

**DOI:** 10.1001/jamanetworkopen.2024.57241

**Published:** 2025-01-30

**Authors:** Jing Liu, Ruchen Zhou, Chengxia Liu, Haiyan Liu, Zhenqin Cui, Zhuang Guo, Weidong Zhao, Xiaoqin Zhong, Xiaodong Zhang, Jing Li, Shihuan Wang, Li Xing, Yusha Zhao, Ruiguang Ma, Jiekun Ni, Zhen Li, Yanqing Li, Xiuli Zuo

**Affiliations:** 1 Department of Gastroenterology, Qilu Hospital of Shandong University, Jinan, Shandong, China; 2 Shandong Provincial Clinical Research Center for Digestive Disease, Jinan, Shandong, China; 3 Laboratory of Translational Gastroenterology, Qilu Hospital of Shandong University, Jinan, Shandong, China; 4 Department of Gastroenterology, Qilu Hospital of Shandong University, Qingdao, Shandong, China; 5 Department of Gastroenterology, Binzhou Medical University Hospital, Binzhou, Shandong, China; 6 Department of Gastroenterology, The First School of Clinical Medicine of Binzhou Medical University, Binzhou, Shandong, China; 7 Department of Gastroenterology, Central Hospital of Shengli Oilfield, Dongying, Shandong, China; 8 Department of Gastroenterology, Zibo Municipal Hospital, Zibo, Shandong, China; 9 Department of Gastroenterology, Linyi People’s Hospital, Dezhou, Shandong, China; 10 Department of Gastroenterology, The People’s Hospital of Zhaoyuan City, Yantai, Shandong, China

## Abstract

**Importance:**

High-quality colonoscopy reduces the risks of colorectal cancer by increasing the adenoma detection rate. Routine use of an automatic quality control system (AQCS) to assist in colorectal adenoma detection should be considered.

**Objective:**

To evaluate the effect of an AQCS on the adenoma detection rate among colonoscopists who were moderate- and low-level detectors during routine colonoscopy.

**Design, Setting, and Participants:**

This multicenter, single-blind, randomized clinical trial was conducted at 6 centers in China from August 1, 2021, to September 30, 2022. Data were analyzed from March 1 to June 30, 2023. Individuals aged 18 to 80 years were enrolled. Exclusion criteria were a history of inflammatory bowel disease, advanced colorectal cancer, and polyposis syndromes; known colorectal polyps without complete removal previously; a history of colorectal surgery; known stenosis or obstruction with contraindication for biopsy or prior failed colonoscopy; pregnancy or lactation; and refusal to participate. Intention-to-treat and per-protocol analysis was used.

**Interventions:**

Standard colonoscopy or AQCS-assisted colonoscopy.

**Main Outcomes and Measures:**

Adenoma detection rate.

**Results:**

A total of 1254 participants (mean [SD] age, 51.21 [12.10] years; 674 [53.7%] male) were randomized (627 standard colonoscopy, 627 AQCS-assisted colonoscopy). Intention-to-treat analysis showed a significantly higher adenoma detection rate in the AQCS-assisted group vs standard colonoscopy group (32.7% vs 22.6%; relative risk [RR], 1.60; 95% CI, 1.23-2.09; *P* < .001). The adenoma detection rates were significantly higher in the AQCS group when considering pathology (nonadvanced adenomas, 30.1% vs 21.2%; RR, 1.52; 95% CI, 1.16-1.99; *P* = .002), and morphology (flat or sessile, 29.3% vs 20.4%, RR, 1.52; 95% CI, 1.16-2.00; *P* = .003). Use of AQCS significantly increased the adenoma detection rate of both the lower-level detectors (30.0% vs 20.0%; RR, 1.71; 95% CI, 1.24-2.35; *P* = .001) and the medium-level detectors (38.1% vs 27.7%; RR, 1.61; 95% CI, 1.07-2.43; *P* = .02). Similar increases were found for adenoma detection rates in the academic and nonacademic centers (academic: 29.3% vs 20.8%; RR, 1.58; 95% CI, 1.10-2.29; *P* = .01; nonacademic: 36.1% vs 24.5%; RR, 1.74; 95% CI, 1.23-2.46; *P* = .002). The number of adenomas per colonoscopy was significantly higher in the AQCS-assisted group (0.86 vs 0.48; RR, 1.50; 95% CI, 1.17-1.91; *P* = .001). The mean withdrawal time without intervention was slightly increased with AQCS assistance (6.78 vs 6.46 minutes; RR, 1.38; 95% CI, 1.26-1.52; *P* < .001). No serious adverse events were reported.

**Conclusions and Relevance:**

In this randomized clinical trial, AQCS assistance during routine colonoscopy increased adenoma detection rates and several related polyp parameters compared with standard colonoscopy in the lower- and medium-level detectors in academic and nonacademic settings. Routine use of AQCS to assist in colorectal adenoma detection and quality improvement should be considered.

**Trial Registration:**

ClinicalTrials.gov Identifier: NCT04901130

## Key Points

**Question:**

Does use of the automatic quality control system (AQCS) affect adenoma detection rates among colonoscopists who were moderate- and low-level detectors during routine colonoscopy?

**Findings:**

In this randomized clinical trial that included 1254 adults, the AQCS-assisted group achieved a significantly higher adenoma detection rate vs the standard colonoscopy group (32.7% vs 22.6%). Adenoma detection rates for nonadvanced adenomas, flat or sessile adenomas, and number of adenomas per colonoscopy were also significantly improved in the AQCS-assisted group.

**Meaning:**

The findings of this study support the application of AQCS to increase the adenoma detection rate of moderate- and low-level detectors in routine colonoscopy practice.

## Teaser

This randomized clinical trial evaluates the use of an automatic quality control system in the detection of colorectal adenomas during colonoscopy.

## Introduction

Colonoscopy efficacy to prevent colorectal cancer (CRC) is widely variable due to the discrepancy of patient-, colonoscopist-, and procedure-related characteristics. In addition, innovation tools for increasing mucosal exposure,^1,2,3^ enhancing imaging,^4,5^ and providing a wider viewing angle^6,7^ are highly dependent on colonoscopist observation. Therefore, quality indicators were introduced, including withdrawal time, cecal intubation, bowel preparation, and photodocumentation of key landmarks to improve colonoscopy quality control.^8,9^ However, these indicators are often not followed well during routine colonoscopies due to colonoscopists’ workload, insufficient immediate supervision, and lack of practical tools.

An automatic quality control system (AQCS) has been developed for the timing of the colonoscopy intubation and withdrawal phase, monitoring withdrawal stability, evaluating bowel preparation, and detecting polyps during high-definition white-light colonoscopy procedures (Video; eFigure 1 in Supplement 1).^10^ The AQCS increased the adenoma detection rate in a randomized clinical trial of 659 participants.^10^ Several studies have shown computer-aided detection (CADe) devices improve the adenoma detection rate using randomized parallel or tandem methods, mainly among Western hemisphere high-performing colonoscopists.^11,12,13,14,15^ This study aimed to evaluate AQCS effectiveness in increasing colorectal adenoma detection rates among colonoscopists who were moderate- and low-level detectors within a Chinese population.

**Video.** AQCS Measures Withdrawal Time, Evaluates Status of Intestinal Lavage, and Displays Polyps in Squares This video of an automatic quality control system (AQCS)–assisted colonoscopy demonstrates the AQCS automatically measuring withdrawal time, evaluating the status of intestinal lavage, and displaying polyps in squares.

## Methods

### Study Design

This parallel, multicenter randomized clinical trial was conducted in 6 centers in China: Qilu Hospital of Shandong University, Jinan; Binzhou Medical University Hospital, Binzhou; Central Hospital of Shengli Oilfield, Dongying; Zibo Municipal Hospital, Zibo; Linyi People’s Hospital, Dezhou; and The People’s Hospital of Zhaoyuan City, Yantai. The protocol (Supplement 2) was approved by the ethics committees of all participating centers. Written informed consent was obtained from all participants; no financial compensation was provided. This report adhered to the Consolidated Standards of Reporting Trials (CONSORT) reporting guideline. The trial was conducted from August 1, 2021, to September 30, 2022, and data were analyzed from March 1 to June 30, 2023.

### Study Population

Individuals aged 18 to 80 years presenting for colonoscopy were enrolled. Exclusion criteria were a history of inflammatory bowel disease, advanced colorectal cancer, and polyposis syndromes; known colorectal polyps without complete removal previously; a history of colorectal surgery; known stenosis or obstruction with contraindication for biopsy or prior failed colonoscopy; pregnancy or lactation; and refusal to participate.

### Randomization and Blinding

All eligible patients were randomly assigned (1:1) to standard colonoscopy or AQCS-assisted colonoscopy via block randomization stratified by center. The randomization lists, generated by R, version 3.6.0 (R Foundation for Statistical Computing), were put into sealed opaque envelopes and then opened at the commencement of the procedure by the staff assistant to ensure blinding of the allocation. Intervention allocation was blinded for patients, pathologists, and statistical analysts, but colonoscopists were aware of group assignment.

### Colonoscopy Procedures

High-definition endoscopy systems (Evis Lucera Elite, Olympus Medical Systems; and EPK-i7000(A)/EPK-i5000/EPK-3000, Pentax Medical) were used. All procedures were performed by high-definition white-light colonoscopes (CF-HQ290, EC-3490TFi, or EC-3890Fi). The endoscopic environment used in the standard colonoscopy group was identical to that in the AQCS group. The time is displayed on the original endoscopic video screen. All procedures were performed by 15 colonoscopists (2-3 for each center; >1500 colonoscopies) (eTable 1 in Supplement 1).^16^ The AQCS monitor (the second monitor) was fixed adjacent and parallel to the original endoscopic video screen. The AQCS was started before intubation and began supervising at withdrawal phases once the cecum was identified. Alongside the original videos, 4 additional visual and audio notices were provided to colonoscopists during the procedure: (1) timer on a second high-definition monitor, (2) prompts for controlling withdrawal speed and reexamining certain segments when unsteady or fuzzy frames were identified continuously by the AQCS, (3) prompts for cleaning mucosa or suctioning liquid pools when suboptimal cleansing (Boston Bowel Preparation Scale [BBPS] score <2) was recognized, and (4) tracking box on the monitor indicating the location of lesions. The preliminary validation data corresponding to the performance level of each function were shown in a previous study.^10^

### Data Collection

Data collected included baseline patient characteristics (age, sex, body mass index, family CRC history, alcohol and tobacco use, and medical history), the procedure details (indication for colonoscopy, sedation, intubation time, withdrawal time without intervention, BBPS score, and basal adenoma detection rate of enrolled colonoscopists), and polyp characteristics (number, size, morphologic characteristics, and location). Basal adenoma detection rate was calculated from 100 consecutive colonoscopies performed by the colonoscopist before study entry. The BBPS score after cleaning was evaluated by experts during the procedure and used as the outcome. The experts were independent and distinct from the colonoscopy operators. All alarm boxes that appeared during the withdrawal phase were recorded and analyzed to see whether they were ultimately false-positive, and colonoscopists were required to carefully check each alarm box (even if only 1 frame was displayed). The number and clinical relevance of false-positive results were also documented.^17^

### Outcome Measures

The primary outcome, adenoma detection rate, was defined as the proportion of individuals with 1 or more adenomas confirmed by histopathologic examination. Sessile serrated lesions were excluded from adenoma detection rate calculation due to variability in pathologist reporting.

Secondary outcomes included advanced adenoma detection rate, proximal colonic adenoma detection rate, adenoma detection rate in academic and nonacademic settings, adenoma detection rate of suboptimal colonoscopy, number of adenomas per colonoscopy (APC), adverse events, and withdrawal time without intervention. The primary analysis was prespecified to be assessed in the intention-to-treat (ITT) population.

### Statistical Analysis 

Based on the mean adenoma detection rate (24%) among patients undergoing colonoscopies at 6 of the participating centers over the past 12 months, a sample size of 501 individuals per group was warranted to yield an 8% increase^18^ in the adenoma detection rate with 80% power (5% α level; 2-sided test) in the AQCS group. Thus, the estimated sample size was 1254 patients in total with a 20% dropout rate.

All data were analyzed according to the ITT population, which included all randomly assigned patients, without imputation or weighting for missing data. Continuous variables are reported as mean (SD) or median (range). Numbers and percentages were used for categorical variables. Baseline clinical and colonoscopy features were compared using the χ^2^ test or Fisher exact test for the categorical variables and the 2-tailed *t* test for the continuous variables. The primary and secondary outcomes were compared by mixed-effects logistic regression or negative binomial regression with the colonoscopist’s level as the random effects, and patient characteristics including indication for colonoscopy (screening, surveillance, and diagnosis), age, sex, smoking history, withdrawal time without intervention, bowel cleanliness, and basal adenoma detection rate levels (<25% and 25%-35%)^12^ as fixed effects. According to the recommendation of American Gastroenterological Association clinical practice,^19^ lower-level detectors were defined as colonoscopists with a basal adenoma detection rate less than 25% and medium-level detectors as those with an adenoma detection rate of 25% to 35%. Accordingly, we fit additional 2-level models without covariates to calculate the intracluster correlation coefficient, which serves as an indicator of the colonoscopist's correlation with the outcome. With 2-sided testing, *P* < .05 was judged as the statistically significant threshold. All data were analyzed with R, version 3.6.0 or higher.

## Results

**Figure 1.**
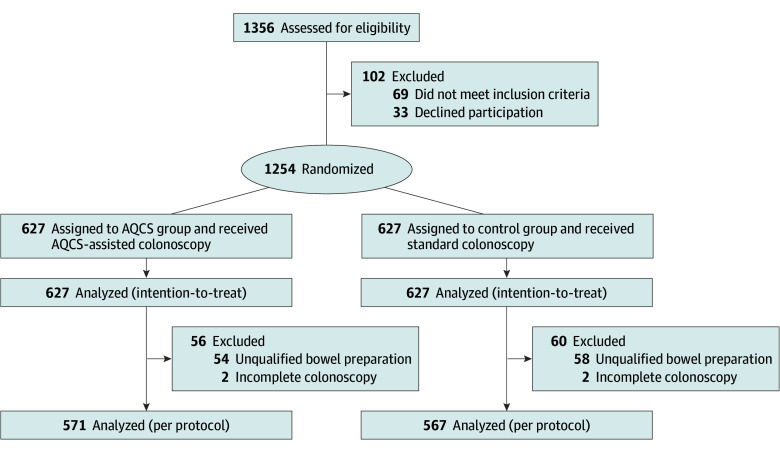
Patient Flowchart AQCS indicates automatic quality control system.

Between August 1, 2021, and September 30, 2022, a total of 1254 participants (mean [SD] age, 51.21 [12.10] years; 580 [46.3%] female; 674 [53.7%] male) were randomly assigned to either standard colonoscopy (n = 627) or AQCS-assisted colonoscopy (n = 627), completed the measures, and were included in ITT analysis (Figure 1). After excluding individuals with incomplete colonoscopy and unqualified bowel preparation, 571 participants in the AQCS-assisted group and 567 in the standard colonoscopy group were included in the per-protocol analysis. Demographic and adenoma risk factors were similar between the groups (Table 1).

**Table 1.** Participants and Colonoscopy Characteristics According to the Intention-to-Treat Analysis

Characteristic	No. (%)	
	AQCS-assisted colonoscopy (n = 627)	Standard colonoscopy (n = 627)
Age, mean (SD), y	50.95 (12.28)	51.46 (11.93)
Sex		
Male	322 (51.4)	352 (56.1)
Female	305 (48.6)	275 (43.9)
BMI, median (IQR)	24.44 (22.46-26.37)	24.57 (22.34-26.37)
Indication		
Screening	208 (33.2)	207 (33.0)
Surveillance	47 (7.5)	49 (7.8)
Diagnosis	372 (59.3)	371 (59.2)
Family CRC history	20 (3.2)	19 (3.0)
Smoking history	171 (27.3)	181 (28.9)
Alcohol use history	243 (38.8)	256 (40.8)
Diabetes	40 (6.4)	34 (5.4)
Hypertension	96 (15.3)	92 (14.7)
Aspirin use	7 (1.1)	9 (1.4)
Procedure time of day		
Morning	307 (49.0)	294 (46.9)
Afternoon or evening	320 (51.0)	333 (53.1)
Anesthesia	469 (74.8)	460 (73.4)
ASA classification		
I	558 (89.0)	558 (89.0)
II	69 (11.0)	69 (11.0)
Initial BBPS, mean (SD)^a^	6.39 (1.62)	6.39 (1.63)
Center of procedure		
Center A	105 (16.7)	104 (16.6)
Center B	104 (16.6)	105 (16.7)
Center C	105 (16.7)	104 (16.6)
Center D	104 (16.6)	105 (16.7)
Center E	105 (16.7)	104 (16.6)
Center F	104 (16.6)	105 (16.7)
Colonoscopist performance		
Lower-level detectors (basal adenoma detection rate <25%)	417 (66.5)	414 (66.0)
Medium-level detectors (basal adenoma detection rate 25%-35%)	210 (33.5)	213 (34.0)

Abbreviations: AQCS, automatic quality control system; ASA, American Society of Anesthesiologists; BBPS, Boston Bowel Preparation Scale; BMI, body mass index (calculated as weight in kilograms divided by height in meters squared); CRC, colorectal cancer.

^a^ Bowel preparation before the cleansing that was scored before further cleansing of the colon lumen during colonoscopy.

The mean overall BBPS score was higher with AQCS assistance, mainly due to improved segmental scores in the right and transverse colon. Both the mean withdrawal time without intervention (6.78 vs 6.46 minutes; difference, 0.32 minutes; relative risk [RR], 1.38; 95% CI, 1.26-1.52; *P* < .001) and the mean withdrawal time for negative colonoscopy (6.65 vs 6.41 minutes; difference, 0.24 minutes; RR, 1.29; 95% CI, 1.16-1.44; *P* < .001) were significantly longer in the AQCS-assisted group. No serious adverse events were reported; 2 patients experienced anesthesia-related hypoxemia (Table 2).

**Table 2.** Findings and Outcomes in AQCS-Assisted and Standard Colonoscopy Groups

Outcome	No. (%)		RR (95% CI)^a^	*P* value
	AQCS-assisted colonoscopy (n = 627)	Standard colonoscopy (n = 627)		
Successful intubation	625 (99.7)	625 (99.7)	1.10 (0.10-12.45)	.94
Bowel preparation adequacy rates^b^	571 (91.4)	567 (90.7)	0.99 (0.66-1.49)	.97
Overall BBPS score, mean (SD)	7.00 (1.52)	6.80 (1.56)	1.21 (1.04-1.39)	.01
Right colon BBPS score, mean (SD)	2.24 (0.63)	2.15 (0.60)	1.09 (1.03-1.17)	.006
Transverse colon BBPS score, mean (SD)	2.40 (0.57)	2.31 (0.58)	1.08 (1.02-1.14)	.007
Left colon BBPS score, mean (SD)	2.36 (0.58)	2.34 (0.61)	1.02 (0.97-1.08)	.40
Intubation time, mean (SD), min	7.62 (6.58)	7.66 (6.15)	0.86 (0.49-1.49)	.58
Withdrawal time without intervention, mean (SD), min	6.78 (1.12)	6.46 (0.97)	1.38 (1.26-1.52)	<.001
Withdrawal time for negative colonoscopy, mean (SD), min	6.65 (0.92)^c^	6.41 (0.97)^d^	1.29 (1.16-1.44)	<.001
Adverse events	1 (0.2)	1 (0.2)	0.99 (0.06-16.31)	>.99
Overall adenoma detection rate	205 (32.7)	142 (22.6)	1.60 (1.23-2.09)	<.001
Adenoma detection rate of suboptimal colonoscopy^e^	21 of 80 (26.3)	12 of 100 (12.0)	2.97 (1.24-7.13)	.02
APC^f^	0.86 (2.19)	0.48 (1.43)	1.50 (1.17-1.91)	.001
Lower-level detectors	0.82 (2.27)	0.34 (1.05)	1.71 (1.24-2.35)	.001
Medium-level detectors	0.94 (2.00)	0.73 (1.94)	1.16 (0.79-1.71)	.45
Academic hospitals	0.62 (1.37)	0.38 (1.17)	1.29 (0.90-1.86)	.16
Nonacademic hospitals	1.09 (2.76)	0.57 (1.64)	1.57 (1.12-2.20)	.009
Screening colonoscopy	0.77 (1.99)	0.48 (1.54)	1.23 (0.80-1.90)	.34
Surveillance colonoscopy	1.57 (3.17)	0.59 (1.15)	1.38 (0.63-3.03)	.43
Diagnostic colonoscopy	0.81 (2.13)	0.46 (1.40)	1.66 (1.18-2.32)	.003
Nonneoplastic lesions per colonoscopy	0.53 (1.35)	0.49 (1.52)	1.01 (0.76-1.35)	.94
USMSTF 1- or 3-y interval, No./total No. (%)	57/618 (9.2)	34/617 (5.5)	1.51 (0.94-2.42)	.09
ESGE 3-y interval, No./total No. (%)	55/618 (8.9)	31/617 (5.0)	1.63 (1.00-2.65)	.05
JGES 1- or 3-y interval, No./total No. (%)	74/619 (12.0)	43/617 (7.0)	1.59 (1.04-2.43)	.03

Abbreviations: AQCS, automatic quality control system; APC, adenomas per colonoscopy; BBPS, Boston Bowel Preparation Scale; ESGE, European Society of Gastrointestinal Endoscopy; JGES, Japan Gastroenterological Endoscopy Society; RR, relative risk; USMSTF, US Multi-Society Task Force on Colorectal Cancer.

^a^ The RRs were computed from mixed-effects logistic or negative binomial regressions, including a random intercept at the colonoscopist level.

^b^ Score of 6 or higher on the BBPS, with no subscore below 2. Data were available for 625 patients in each group.

^c^ n = 327.

^d^ n = 378.

^e^ Unqualified bowel preparation, withdrawal time less than 6 minutes, or incomplete colonoscopy.

^f^ Lower-level detectors (basal adenoma detection rate <25%; n = 10) performed 417 colonoscopies in the AQCS group and 414 in the standard colonoscopy group. Medium-level detectors (basal adenoma detection rate 25%-35%; n = 5) performed 210 colonoscopies in the AQCS group and 213 in the standard colonoscopy group. APC was stratified by indication of screening (n = 415), surveillance (n = 96), or diagnosis (n = 743).

**Figure 2.**
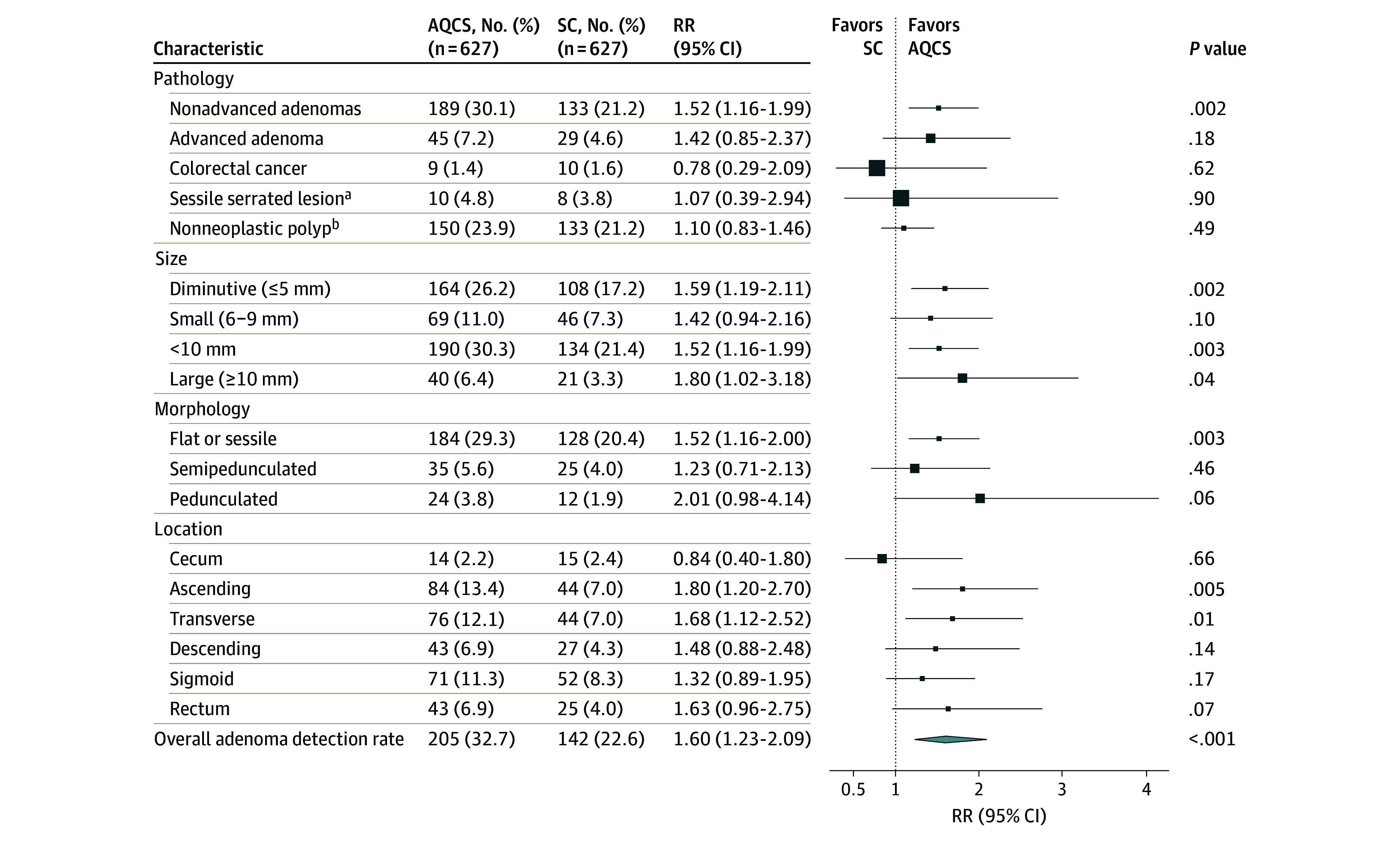
Comparison of the Adenoma Detection Rate Between Automatic Quality Control System (AQCS) and Standard Colonoscopy (SC) Groups According to Intention-to-Treat Analysis RR indicates relative risk. The sizes of the boxes represent the magnitude of the SE. ^a^Sessile serrated lesion was not included in the detection rate of adenoma. Data available for 209 patients in each group. ^b^Polyps with histologic testing showing hyperplastic, inflammatory lesions, normal mucosa, and others.

The adenoma detection rate was 32.7% (205 of 627) in the AQCS group and 22.6% (142 of 627) in the standard colonoscopy group (RR, 1.60; 95% CI, 1.23-2.09; *P* < .001; intracluster correlation coefficient, <0.001) (Table 2). AQCS implementation resulted in a 10.1–percentage point increase in the adenoma detection rate compared to with the standard colonoscopy group (22.6%). Per-protocol analysis results were consistent with the ITT analysis results. In addition, the adenoma detection rate of suboptimal colonoscopy was significantly higher in the AQCS than in the standard colonoscopy group (26.3% vs 12.0%; *P* = .02). Significant differences were found only in the histologic evaluation subgroups with nonadvanced adenomas (Figure 2). The detection rate of nonneoplastic polyps was similar between groups. Of these, patients with no pathologically proven adenoma, CRCs, or sessile serrated lesions after polypectomy accounted for 11.8% (74 of 627) in the AQCS group and 13.7% (86 of 627) in the standard colonoscopy group (RR, 0.95; 95% CI, 0.67-1.36; *P* = .78). The detection rate of adenomas was significantly higher in the AQCS group when considering shape (flat or sessile), size (diminutive and large), and location (ascending and transverse colon). The proportional effect of AQCS assistance on the adenoma detection rate was consistent across all 8 subgroups (all *P* < .05 for interaction) (eFigure 2 in Supplement 1), including by center characteristics and colonoscopist performance. Compared with standard colonoscopy group, use of AQCS significantly increased the adenoma detection rate of both the lower-level detectors (30.0% vs 20.0%; RR, 1.71; 95% CI, 1.24-2.35; *P* = .001) and the medium-level detectors (38.1% vs 27.7%; RR, 1.61; 95% CI, 1.07-2.43; *P* = .02). Similar increases were found for adenoma detection rates in the academic and nonacademic centers (academic: 29.3% vs 20.8%; RR, 1.58; 95% CI, 1.10-2.29; *P* = .01; nonacademic: 36.1% vs 24.5%; RR, 1.74; 95% CI, 1.23-2.46; *P* = .002). The adenoma detection rate increase with AQCS was 8.5% in academic centers and 11.6% in nonacademic centers.

The number of polyps per colonoscopy were 1.39 the AQCS group and 0.96 in the standard colonoscopy group (RR, 1.23; 95% CI, 1.01-1.51) (eTable 2 in Supplement 1). The APC in the AQCS group was higher than that in the standard colonoscopy group (0.86 vs 0.48; RR, 1.50; 95% CI, 1.17-1.91; *P* = .001) (Table 2). The APC in the AQCS group was higher than that in the standard colonoscopy group among the lower-level detectors (0.82 vs 0.34; RR, 1.71; 95% CI, 1.24-2.35; *P* = .001), but not among the medium-level detectors (0.94 vs 0.73; RR, 1.16; 95% CI, 0.79-1.71). The AQCS offered the benefit of APC for the nonacademic centers (1.09 vs 0.57; RR, 1.57; 95% CI, 1.12-2.20), but not for the academic centers (0.62 vs 0.38; RR, 1.29; 95% CI, 0.90-1.86). Use of AQCS improved the APC for diagnostic colonoscopy (0.81 vs 0.46; RR, 1.66; 95% CI, 1.18-2.32). A statistically significant increase in the APC with use of AQCS was found for flat or sessile lesions, proximal colonic lesions, lesions less than 10 mm, and nonadvanced adenomas, but not for the mean number of advanced adenomas or nonneoplastic polyps (eTable 3 in Supplement 1).

The adenoma detection rate was significantly increased with the assistance of AQCS (RR, 1.60; 95% CI, 1.23-2.09; *P* < .001) (Table 3). Factors contributing to a higher adenoma detection rate included surveillance colonoscopy, increased withdrawal time without intervention, qualified BBPS score, advanced age, and male sex. Additionally, advanced age, longer withdrawal time without intervention, and smoking history increased the adenoma detection rate, whereas AQCS, qualified BBPS score, and colonoscopist performance did not have a notable influence on the adenoma detection rate.

**Table 3.** Multivariate Regression Analysis of Predictors of Adenoma and Advanced Adenoma Detection Rate^a^

Variable	Adenoma detection rate^b^		Advanced adenoma detection rate^c^	
	RR (95% CI)	*P* value	RR (95% CI)	*P* value
AQCS arm	1.60 (1.23-2.09)	<.001	1.42 (0.85-2.37)	.18
Indication				
Surveillance colonoscopy (screening colonoscopy as reference)	1.67 (1.02-2.74)	.04	0.55 (0.17-1.76)	.31
Diagnostic colonoscopy (screening colonoscopy as reference)	0.99 (0.74-1.33)	.97	1.07 (0.57-2.02)	.83
Smoking history	1.33 (0.95-1.86)	.10	2.01 (1.04-3.89)	.04
Age, y	1.05 (1.04-1.06)	<.001	1.07 (1.05-1.10)	<.001
Male	1.41 (1.03-1.93)	.03	1.41 (0.73-2.72)	.30
Withdrawal time without intervention, min	1.25 (1.09-1.42)	.001	1.55 (1.24-1.94)	<.001
Qualified BBPS	2.79 (1.63-4.78)	<.001	1.38 (0.60-3.15)	.45
Colonoscopist performance: medium-level detectors^d^	1.22 (0.90-1.64)	.20	1.67 (0.68-4.11)	.26

Abbreviations: AQCS, automatic quality control system; BBPS, Boston Bowel Preparation Scale; RR, relative risk.

^a^ Results from a 2-level logistic regression model with adenoma detection as the outcome and patient characteristics (fixed effects) and colonoscopist performance (random effect) as the regression.

^b^ Between-colonoscopist SD (null model): estimate, <0.001 (95% CI, 0.00-0.24); intraclass correlation coefficient, <0.001.

^c^ Between-colonoscopist SD (null model): estimate, 0.72 (95% CI, 0.36-1.32); intraclass correlation coefficient, 0.14.

^d^ Medium-level detectors were 5 colonoscopists with a mean basal adenoma detection rate of 25% to 35%. Lower-level detectors (as reference) were 10 colonoscopists with a mean basal adenoma detection rate of less than 25%.

According to the 2020 US Multi-Society Task Force guideline on colonoscopy surveillance,^20^ the proportion of patients who are recommended to have 1- and 3-year surveillance intervals increased from 5.5% in the standard colonoscopy group to 9.2% in the AQCS group (absolute difference, 3.7 percentage points) (Table 2). With use of the Japan Gastroenterological Endoscopy Society guideline,^21^ AQCS significantly increased the proportion of patients recommended to use a 1- or 3-year surveillance interval within the lower-level detector groups (eTable 4 in Supplement 1).

A total of 809 false-positive detections were registered across the 623 videos of the AQCS group, corresponding to a mean of 1.3 false-positive detections per colonoscopy. Among them, 0.9 false-positive detections (72.6%) per colonoscopy were due to folds and other artifacts from the bowel wall. Conversely, 0.4 false-positive detections (27.4%) were caused by artifacts from bowel content. In detail, 515 false-positive detections (63.7%) were classified as being of no clinical relevance (0 seconds), 255 as mild clinical relevance (≤1 second) (31.5%), 36 as moderate clinical relevance (1-3 seconds) (4.4%), and 3 as severe clinical relevance (>3 seconds) (0.4%). No false-positive detections triggered biopsy or polypectomy.

## Discussion

In this multicenter randomized clinical trial, a multifaceted quality improvement intervention using AQCS effectively improved the adenoma detection rate of moderate- and low-level detectors during routine colonoscopy in both academic and nonacademic settings. The mixed-effects regression model identified that AQCS-assisted colonoscopy significantly improved the adenoma detection rate, polyp detection rate, and APC for routine procedures after adjustment for possible confounders, including the variability between patients and colonoscopists. The positive results were accompanied by a slightly prolonged withdrawal time (6.65 vs 6.41 minutes. difference, 0.24minutes) for negative colonoscopy. The AQCS-assisted colonoscopy offered benefits for both moderate- and low-level detectors in both the academic and nonacademic settings and could even attenuate the influence of suboptimal colonoscopy (unqualified bowel preparation, withdrawal time <6 minutes, or incomplete colonoscopy) on the adenoma detection rate.

The role of CADe assistance in colonoscopy for colonoscopists with varying proficiency levels remains unclear. In a recent study,^12^ while the difference was not significant, the lower detectors (basal adenoma detection rate <25%) might achieve higher improvement in the adenoma detection rate (6.2%) with CADe assistance, compared with the medium-level detectors (3.7%). Another study reported that experts (≥5000 colonoscopies) benefit more from artificial intelligence–assisted colonoscopy,^22^ while an Italian study showed similar benefits across colonoscopists with varying experience levels.^23^ Such inconsistencies may result from adenoma detection rate variability among colonoscopists and patient-dependent factors.^24^ In our study, AQCS improved adenoma detection rates similarly across both medium-level (10.4%) and lower-level (10.0%) detectors, suggesting that experience may not be essential for adenoma detection rate improvement with AQCS. This is probably because of AQCS supervision of bowel cleanliness and withdrawal time improving mucosal visibility and reducing inattentional blindness. Even with the assistance of AQCS, the adenoma detection rate in low-level detectors (30.0%) was still lower than that in medium-level detectors (38.1%), indicating that other objective withdrawal techniques focused on behind-fold inspection and distension are warranted for higher adenoma detection rates.^25,26,27^

In our study, AQCS implementation resulted in a 10.1% increase in the adenoma detection rate compared with the standard colonoscopy group (22.6%), similar to a 10.3% increase reported in a meta-analysis of 5 randomized clinical trials in China.^28^ The adenoma detection rate increase with AQCS was 8.5% in academic centers and 11.6% in nonacademic centers in our study. While artificial intelligence–assisted colonoscopy has shown benefits in university-affiliated or military endoscopy centers,^22,29^ evidence from nonacademic centers is limited and inconsistent.^30,31,32^ In a French study with 2039 patients,^12^ CADe increased the adenoma detection rate from 33.7% to 37.5% (*P* = .05) during routine colonoscopy in a nonacademic center. A US study in nonacademic centers found a small, but nonsignificant, benefit in the adenoma detection rate of 3.9% from a baseline rate of 43.9% and APC of 0.22 from a baseline mean of 0.83 (*P* = .002) in a relatively lower risk population (screening and surveillance only) by high-performing colonoscopists (baseline adenoma detection rate, 43.9%).^31^ Another US study in 4 community-based centers found that CADe was not superior to standard colonoscopy for the adenoma detection rate (35.9% vs 37.2%; *P* = .77).^32^ The adenoma detection rate in our study is lower than reported in studies from European countries and the US.^33^ This may be largely due to the relatively lower CRC risk population recruited in our study. Nevertheless, this low adenoma detection rate may limit the generalizability of our findings.

The relatively higher improvement in the adenoma detection rate at nonacademic settings observed in our trial (11.6%) could be partly attributed to the lower baseline adenoma detection rate of 22.6% among routine colonoscopies, compared with aforementioned studies (ranging from 33.7% to 43.9%),^12,31,32^ which were limited to European and US populations and found much smaller benefits, and even negative outcomes, of CADe. Also noteworthy is the participating colonoscopists with low to moderate performance (adenoma detection rate <35%), leaving room for more improvement in adenoma detection.^12^

Our findings also support the efficacy of AQCS on APC and polyp detection rate, which corresponded to available evidence.^34,35,36^ The APC clinical improvement is important in identifying high- and low-quality procedures and is recognized as a more granular measure to discriminate colonoscopists with similar adenoma detection rates.^37,38^ The discriminatory benefit of APC was observed among low-level detectors in our study, but not medium-level detectors. When stratified by the indication of colonoscopy, the APC was not significantly different between screening and surveillance procedures, but differences were noted only in diagnostic procedures. This suggests that relevant factors beyond AQCS may influence the APC and that its added values might be restricted in a subset of indications. Unlike other published data,^22^ there was no significant difference in the overall adenoma detection rate in this study, probably because most advanced adenomas are large, which is not the strength of AQCS. By contrast, the adenoma detection rate improvement by AQCS is met with an increased proportion of patients requiring intensive postpolypectomy surveillance, with absolute increases of 3.7% (US Multi-Society Task Force guideline) and 3.9% (European Society of Gastrointestinal Endoscopy guideline). The patients with AQCS-upshifted high-risk adenoma were more common among low-level detectors by the Japan Gastroenterological Endoscopy Society guideline. Surveillance strategies are warranted to offset the risk of overdiagnosis and CRC prevention effect.^39^

In addition, the adenoma detection rate of suboptimal colonoscopy was significantly higher in the AQCS than in the standard colonoscopy group (26.3% vs 12.0%; *P* = .02). This finding indicated the AQCS might improve mucosal visibility by reminding the detectors of cleansing maneuvers. However, although the difference between the BBPS in the AQCS and standard colonoscopy groups was statistically significant, it may not be clinically relevant. The time used to reach a slight improvement in the BBPS may not impact colonoscopy performance but only the withdrawal time, which was slightly longer in the AQCS group.

Regarding harms, mean nonneoplastic lesions per colonoscopy were similar between the AQCS and standard colonoscopy groups. This suggests that colonoscopists may have visually diagnosed and subsequently disregarded nonneoplastic polyps. A slightly prolonged withdrawal time without intervention was identified in the AQCS group, most likely owing to more time to clean the mucosa or suction liquid pools when an inadequate score was given by the AQCS. In addition, false-positive detections may hamper the effectiveness of AQCS by prolonging mucosal examination time and frustrating colonoscopists to use this tool. In this study, only about 5% of false alarms lasted for more than 1 second, which may have some clinical relevance. Furthermore, no immediate or delayed adverse events were observed.

### Strengths and Limitations

Strengths of our study include multiple academic and nonacademic settings, stratified block randomization, the adaptability of equipment (Olympus and Pentax), and lower- and medium-level detectors. All of these factors reflect current routine clinical practice.

This study has certain limitations. First, potential Hawthorne bias might exist due to the inability to blind the colonoscopists to the patient assignment. The exact benefit of the AQCS may be overrated because detectors may be more attentive when under observation. Second, the association between adenoma findings by AQCS and long-term data, including CRC and cost-effectiveness, is unclear. A recent study using a Markov microsimulation model suggests that the adoption of artificial intelligence in screening scenarios is a cost-saving strategy to additionally prevent 7194 CRC cases and 2089 related deaths per year.^39^ Third, AQCS does not implement blind spot recognition and reminders. Fourth, the absence of a third control arm that includes only the CADe limits the ability to draw conclusions about the real advantage of AQCS over CADe. However, previous studies have evaluated the use of CADe in improving the adenoma detection rate, and the effect of colonoscopy quality control on adenoma detection rate is well established.^11,12,13,14,15,33^ Fifth, the synergistic effect of interventions, including mucosal exposure devices, image-enhanced technologies, and short-retraining courses, may be reasonably considered and further investigated.

## Conclusions

In our multicenter randomized clinical trial, AQCS assistance during routine colonoscopy improved the adenoma detection rate, APC, and adenoma detection rate of both low- and medium-level examiners in academic and nonacademic settings. A possible association between adenoma findings by AQCS and long-term data, including CRC and cost-effectiveness, needs further investigation.

## Notes

Supplement 1. **eFigure 1.** The Appearance of AQCS **eFigure 2.** Subgroup Analysis of the Adenoma Detection Rate by Intention-to-Treat Analysis **eTable 1.** The Distribution of Colonoscopists’ Performance (Lower- or Medium-Level Detector) at Each Center **eTable 2.** Other Findings and Outcomes in AQCS-Assisted and SC Groups **eTable 3.** (Incident) Rate Ratios (IRR) and 95% Confidence Intervals for the Multilevel Negative Binomial Regression Model **eTable 4.** Subgroup Analysis of Surveillance Recommendation by Colonoscopists’ Performance and Hospital Characteristics, Comparing AQCS–Assisted Group With SC Group

Supplement 2.
Trial Protocol

Supplement 3.
Data Sharing Statement
