# Supplementary material for: Automatic Quality Control System and Adenoma Detection Rates During Routine Colonoscopy: A Randomized Clinical Trial
Source: JAMA Netw Open. 2025 Jan 30;8(1):e2457241. doi: 10.1001/jamanetworkopen.2024.57241 (PMC11783196; doi:10.1001/jamanetworkopen.2024.57241)
Supplement: Supplement 1. — eFigure 1. The Appearance of AQCS eFigure 2. Subgroup Analysis of the Adenoma Detection Rate by Intention-to-Treat Analysis eTable 1. The Distribution of Colonoscopists’ Performance (Lower- or Medium-Level Detector) at Each Center eTable 2. Other Findings and Outcomes in AQCS-Assisted and SC Groups eTable 3. (Incident) Rate Ratios (IRR) and 95% Confidence Intervals for the Multilevel Negative Binomial Regression Model eTable 4. Subgroup Analysis of Surveillance Recommendation by Colonoscopists’ Performance and Hospital Characteristics, Comparing AQCS–Assisted Group With SC Group [file jamanetwopen-e2457241-s001.pdf]

Supplementary Online Content

Liu J, Zhou R, Liu C, et al. Automatic quality control system and adenoma detection rates during routine colonoscopy: a randomized clinical trial. *JAMA Netw Open*. 2025;8(1):e2457241. doi:10.1001/jamanetworkopen.2024.57241

eFigure 1. The Appearance of AQCS

eFigure 2. Subgroup Analysis of the Adenoma Detection Rate by Intention-to-Treat Analysis

eTable 1. The Distribution of Colonoscopists' Performance (Lower- or Medium-Level Detector) at Each Center

eTable 2. Other Findings and Outcomes in AQCS-Assisted and SC Groups

eTable 3. (Incident) Rate Ratios (IRR) and 95% Confidence Intervals for the Multilevel Negative Binomial Regression Model

eTable 4. Subgroup Analysis of Surveillance Recommendation by Colonoscopists' Performance and Hospital Characteristics, Comparing AQCS–Assisted Group With SC Group

This supplementary material has been provided by the authors to give readers additional information about their work.

© 2025 Liu J et al. *JAMA Network Open.*

eFigure 1. The appearance of AQCS.

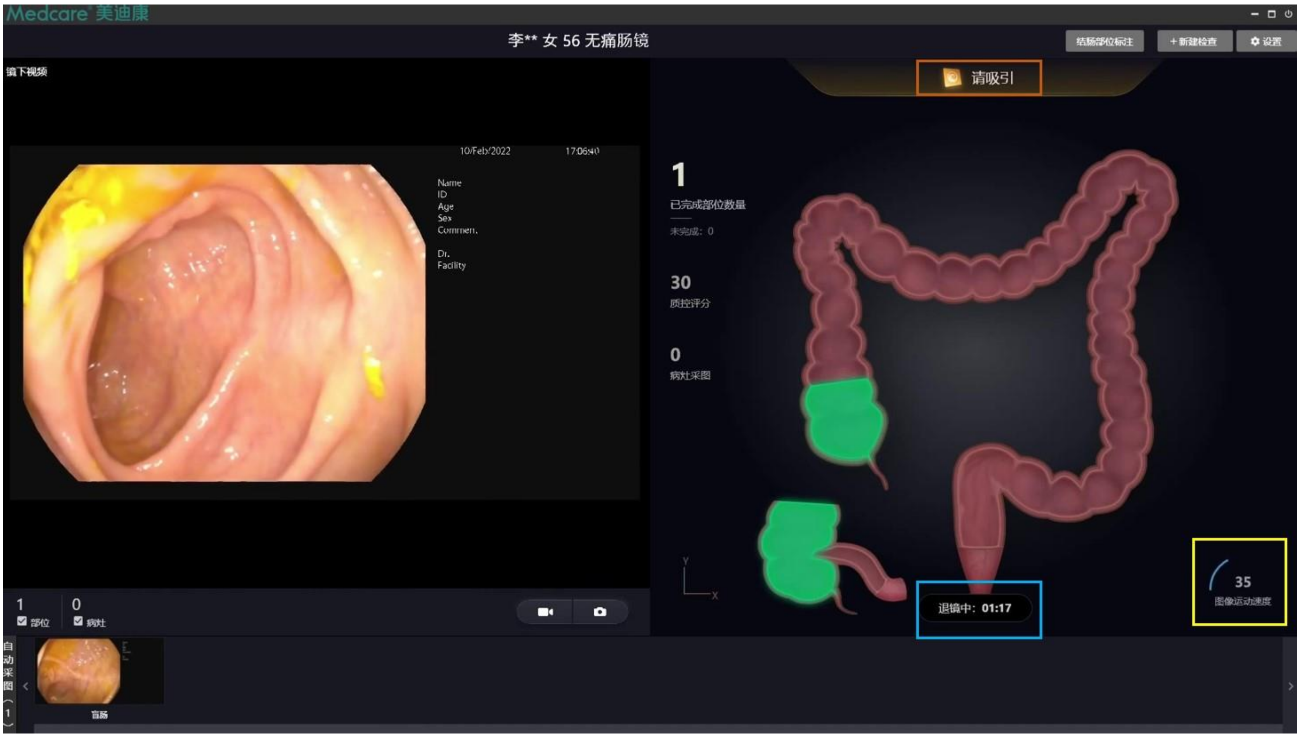

Alongside the original videos, four additional visual and audio notices were provided to endoscopists in real-time: (1) timer on a second high-definition monitor (hollow blue box); (2) prompts for controlling withdrawal speed and reexamining certain segments when unsteady or fuzzy frames identified continuously by AQCS (hollow yellow box); (3) prompts for cleaning mucosa or suctioning liquid pools when suboptimal cleansing (BBPS score <2) was recognised (hollow orange box) and (4) tracking box on the monitor indicating lesions location (eVideo).

© 2025 Liu J et al. *JAMA Network Open.*

eFigure 2. Subgroup analysis of the adenoma detection rate by intention-to-treat analysis.

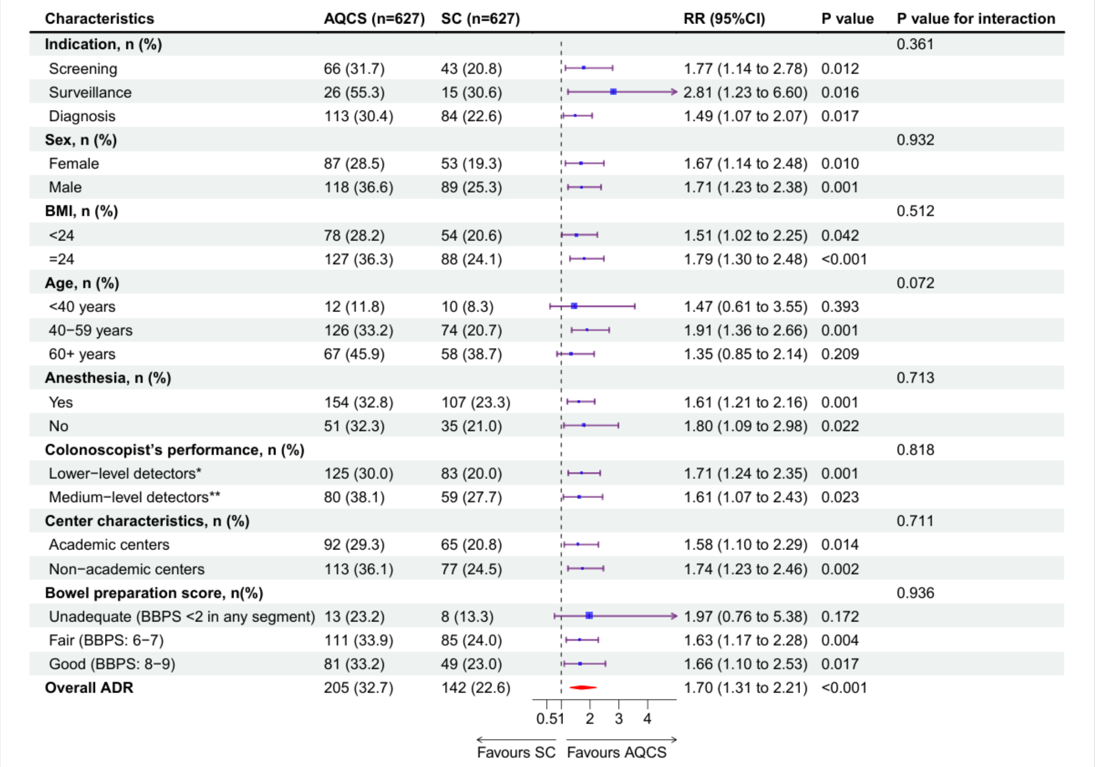

Characteristics	AQCS (n=627)	SC (n=627)	RR (95%CI)	P value	P value for interaction
**Indication, n (%)**					0.361
Screening	66 (31.7)	43 (20.8)	1.77 (1.14 to 2.78)	0.012	
Surveillance	26 (55.3)	15 (30.6)	2.81 (1.23 to 6.60)	0.016	
Diagnosis	113 (30.4)	84 (22.6)	1.49 (1.07 to 2.07)	0.017	
**Sex, n (%)**					0.932
Female	87 (28.5)	53 (19.3)	1.67 (1.14 to 2.48)	0.010	
Male	118 (36.6)	89 (25.3)	1.71 (1.23 to 2.38)	0.001	
**BMI, n (%)**					0.512
<24	78 (28.2)	54 (20.6)	1.51 (1.02 to 2.25)	0.042	
=24	127 (36.3)	88 (24.1)	1.79 (1.30 to 2.48)	<0.001	
**Age, n (%)**					0.072
<40 years	12 (11.8)	10 (8.3)	1.47 (0.61 to 3.55)	0.393	
40−59 years	126 (33.2)	74 (20.7)	1.91 (1.36 to 2.66)	0.001	
60+ years	67 (45.9)	58 (38.7)	1.35 (0.85 to 2.14)	0.209	
**Anesthesia, n (%)**					0.713
Yes	154 (32.8)	107 (23.3)	1.61 (1.21 to 2.16)	0.001	
No	51 (32.3)	35 (21.0)	1.80 (1.09 to 2.98)	0.022	
**Colonoscopist's performance, n (%)**					0.818
Lower−level detectors*	125 (30.0)	83 (20.0)	1.71 (1.24 to 2.35)	0.001	
Medium−level detectors**	80 (38.1)	59 (27.7)	1.61 (1.07 to 2.43)	0.023	
**Center characteristics, n (%)**					0.711
Academic centers	92 (29.3)	65 (20.8)	1.58 (1.10 to 2.29)	0.014	
Non−academic centers	113 (36.1)	77 (24.5)	1.74 (1.23 to 2.46)	0.002	
**Bowel preparation score, n(%)**					0.936
Unadequate (BBPS <2 in any segment)	13 (23.2)	8 (13.3)	1.97 (0.76 to 5.38)	0.172	
Fair (BBPS: 6−7)	111 (33.9)	85 (24.0)	1.63 (1.17 to 2.28)	0.004	
Good (BBPS: 8−9)	81 (33.2)	49 (23.0)	1.66 (1.10 to 2.53)	0.017	
**Overall ADR**	205 (32.7)	142 (22.6)	1.70 (1.31 to 2.21)	<0.001	

AQCS, automatic quality control system; SC, standard colonoscopy; RR, relative risk; CI, confidence interval; BMI, body mass index; BBPS, Boston bowel preparation score; ADR, adenoma detection rate. *Lower-level detectors were ten colonoscopists with a mean basal ADR of less than 25%. **Medium-level detectors were five colonoscopists with a mean basal ADR of 25%–35%.

© 2025 Liu J et al. *JAMA Network Open.*

eTable 1. The distribution of colonoscopists' performance (lower- or medium-level detector) at each center.

	Lower-level detector (n=10)	Medium-level detector (n=5)
Center A	3	0
Center B	2	0
Center C	2	0
Center D	0	2
Center E	1	2
Center F	2	1

* Lower-level detectors (as reference) were ten colonoscopists with a mean basal ADR of less than 25%.

** Medium-level detectors were five colonoscopists with a mean basal ADR of 25–35%.

© 2025 Liu J et al. *JAMA Network Open.*

eTable 2. Other findings and outcomes in AQCS-assisted and SC groups

	AQCS-assisted group (n=627)	SC group (n=627)	RR (95%CI)	*P* value
Polyp detection rate	283 (45·1)	230 (36·7)	1·34 (1·05-1·70)	0·02
Polyps per colonoscopy	1·39 (2·69)	0·96 (2·19)	1·23 (1·01-1·51)	0·04
Advanced ADR	45 (7·2)	29 (4·6)	1·42 (0·85-2·37)	0·18
Advanced ADR of lower-level detectors*	23/417 (5·5)	11/414 (2·7)	1·53 (0·69-3·37)	0·29
Advanced ADR of medium-level detectors**	22/210 (10·5)	18/213 (8·5)	1·22 (0·61-2·42)	0·57
Advanced ADR of academic hospitals	11/314 (3·5)	8/313 (2·6)	0·76 (0·25-2·36)	0·64
Advanced ADR of non-academic hospitals	34/313 (10·9)	21/314 (6·7)	1·60 (0·88-2·91)	0·13
Advanced adenomas per colonoscopy	0·11 (0·51)	0·07 (0·41)	1·38 (0·81-2·35)	0·23

Values are n (%), mean (SD), or n/n (%). The RRs were computed from mixed-effects logistic or negative binomial regressions, including a random intercept at the colonoscopist level.

AQCS, automatic quality control system; SC, standard colonoscopy; ADR, adenoma detection rate.

* Lower-level detectors (as reference) were ten colonoscopists with a mean basal ADR of less than 25%.

** Medium-level detectors were five colonoscopists with a mean basal ADR of 25–35%.

© 2025 Liu J et al. *JAMA Network Open.*

eTable 3. (Incident) Rate ratios (IRR) and 95% confidence intervals for the multilevel negative binomial regression model. ICC (intraclass correlation coefficient) for null multilevel model

	APC for entire colon			APC for flat or sessile lesions			APC for proximal colonic lesions			APC for lesions < 10mm		
**Variables**	**IRR**	**95% CI**	***P* value**	**IRR**	**95% CI**	***P* value**	**IRR**	**95% CI**	***P* value**	**IRR**	**95% CI**	***P* value**
Study arm-AQCS	1·50	1·17-1·91	0·001	1·49	1·15-1·93	0·002	1·56	1·14-2·14	0·006	1·46	1·13-1·88	0·004
Indication												
Surveillance colonoscopy (screening colonoscopy as reference)	1·40	0·89-2·20	0·14	1·49	0·94-2·36	0·09	1·08	0·60-1·92	0·80	1·53	1·13-1·88	0·07
Diagnostic colonoscopy (screening colonoscopy as reference)	1·10	0·84-1·45	0·47	1·11	0·84-1·47	0·46	0·95	0·68-1·34	0·79	1·10	0·97-2·41	0·51
Smoking history	1·15	0·84-1·56	0·38	1·04	0·75-1·43	0·82	0·93	0·62-1·38	0·71	1·03	0·83-1·45	0·86
Age (years)	1·05	1·04-1·06	<0·001	1·05	1·03-1·06	<0·001	1·05	1·03-1·06	<0·001	1·05	1·03-1·06	<0·001
Male	1·77	1·32-2·36	<0·001	1·78	1·32-2·40	<0·001	1·87	1·29-2·70	<0·001	1·80	1·34-2·42	<0·001
Withdrawal time without intervention, min	1·50	1·33-1·68	<0·001	1·55	1·37-1·75	<0·001	1·61	1·38-1·87	<0·001	1·53	1·35-1·73	<0·001
Qualified BBPS	1·69	1·09-2·63	0·02	1·67	1·04-2·66	0·03	1·38	0·79-2·41	0·25	1·70	1·08-2·70	0·02

© 2025 Liu J et al. *JAMA Network Open.*

Colonoscopist's performance–medium-level detectors**	1·00	0·76-1·31	0·999	0·84	0·63-1·11	0·22	0·86	0·61-1·22	0·41	0·93	0·70-1·22	0·59
**Random part**	**Estimate**	**95% CI**		**Estimate**	**95% CI**		**Estimate**	**95% CI**		**Estimat e**	**95% CI**	
Between colonoscopist standard deviation (null model)	0·42	0·25-0·69		0·41	0·24-0·69		0·45	0·25-0·76		0·44	0·26-0·73	
ICC	0·15			0·15			0·17			0·16		

	**APC for advanced adenoma**			**APC for non-advanced adenoma**			**APC for non-neoplastic polyp***		
**Variables**	**IRR**	**95% CI**	***P* value**	**IRR**	**95% CI**	***P* value**	**IRR**	**95% CI**	***P* value**
Study arm-AQCS	1·38	0·81-2·35	0·23	1·48	1·15-1·91	0·003	1·01	0·76-1·35	0·94
Indication									
Surveillance colonoscopy (screening colonoscopy as reference)	0·86	0·30-2·46	0·78	1·45	0·92-2·29	0·11	1·33	0·78-2·26	0·29
Diagnostic colonoscopy (screening colonoscopy as reference)	1·29	0·67-2·48	0·44	1·06	0·80-1·39	0·69	0·86	0·61-1·21	0·40
Smoking history	1·78	0·90-3·53	0·01	1·08	0·78-1·48	0·65	1·06	0·73-1·55	0·75

© 2025 Liu J et al. *JAMA Network Open.*

Age (years)	1·07	1·04-1·10	<0·001	1·05	1·03-1·06	<0·001	1·02	1·01-1·03	<0·001
Male	1·96	0·99-3·86	0·05	1·74	1·30-2·34	<0·001	1·20	0·86-1·67	0·29
Withdrawal time without intervention, min	1·39	1·11-1·74	0·004	1·54	1·36-1·73	<0·001	1·45	1·17-1·80	<0·001
Qualified BBPS	0·89	0·41-1·96	0·78	2·00	1·24-3·24	0·005	1·18	0·72-1·93	0·51
Colonoscopist's performance–medium-level detectors**	2·58	1·25-5·32	0·01	0·87	0·66-1·15	0·31	0·83	0·31-2·24	0·71
**Random part**	**Estimate**	**95% CI**		**Estimate**	**95% CI**		**Estimate**	**95% CI**	
Between colonoscopist standard deviation (null model)	0·94	0·54-1·65		0·45	0·26-0·73		0·67	0·43-1·09	
ICC	0·47			0·17			0·31		

APC, adenomas per colonoscopy; IRR, incident rate ratios; CI, confidence interval; AQCS, automatic quality control system; BBPS, Boston Bowel Preparation Scale; ICC, intraclass correlation coefficient. *Polyps with histology showing hyperplastic, inflammatory lesions, normal mucosa and others. ** Medium-level detectors were five colonoscopists with a mean basal ADR of 25–35%. Lower-level detectors (as reference) were ten colonoscopists with a mean basal ADR of less than 25%.

© 2025 Liu J et al. *JAMA Network Open.*

eTable 4. Subgroup analysis of surveillance recommendation by colonoscopists' performance and hospital characteristics, comparing AQCS–assisted group with SC group

	**USMSTF (1 or 3-year interval)**		**ESGE (3-year interval)**		**JGES (1 or 3-year interval)**	
	**RR**	**95% CI**	**RR**	**95% CI**	**RR**	**95% CI**
All	1·51	0·94-2·42	1·63	1·00-2·65	1·59	1·04-2·43
Colonoscopist's performance						
Lower-level detectors (basal ADR <25%)	1·67	0·84-3·32	1·89	0·92-3·89	1·98	1·08-3·64
Medium-level detectors (basal ADR 25-35%)	1·22	0·62-2·38	1·30	0·66-2·56	1·13	0·61-2·10
Hospital characteristics						
Academic hospitals	0·77	0·31-1·95	0·93	0·35-2·46	1·06	0·48-2·37
Non-academic hospitals	1·72	0·96-3·09	1·81	0·99-3·27	1·57	0·92-2·68

The RRs were computed from mixed-effects logistic or negative binomial regressions, including a random intercept at the colonoscopist level.

AQCS, automatic quality control system; SC, standard colonoscopy; USMSTF, US Multi-Society Task Force on Colorectal Cancer; ESGE, European Society of Gastrointestinal Endoscopy; JGES, Japan Gastroenterological Endoscopy Society; RR, relative risk; CI, confidence interval; ADR, adenoma detection rate.

© 2025 Liu J et al. *JAMA Network Open.*
