# Supplementary material for: Automatic Quality Control System and Adenoma Detection Rates During Routine Colonoscopy: A Randomized Clinical Trial
Source: JAMA Netw Open. 2025 Jan 30;8(1):e2457241. doi: 10.1001/jamanetworkopen.2024.57241 (PMC11783196; doi:10.1001/jamanetworkopen.2024.57241)
Supplement: Supplement 2. — Trial Protocol [file jamanetwopen-e2457241-s002.pdf]

Effect of Automatic Quality Control System on Adenoma Detection Rates During Routine Colonoscopy: a multicenter randomized clinical trial

Version: 2.0

Date: 27-Mar-2021

Table of content

1. **Committees**
2. **Introduction**
3. **Objectives**
4. **Outcomes**
5. **Study design**
6. **Study population**
7. **Study procedures**
8. **Assessment of safety**
9. **Data analysis**
10. **Ethics**
11. **Data quality control**
12. **Data share**

1. Committees

1) Principal investigators: Yan-Qing Li, M.D., Ph.D.

Department of Gastroenterology, Qilu Hospital of Shandong University, Jinan, Shandong, China; Laboratory of Translational Gastroenterology, Qilu Hospital of Shandong University, Jinan, Shandong, China; Robot engineering laboratory for precise diagnosis and therapy of GI tumor, Qilu Hospital of Shandong University, Jinan, Shandong, China.

2) Steering Committee

Name	Affiliation	Email address
Yan-Qing Li	Department of Gastroenterology, Qilu Hospital of Shandong University	liyanqing@sdu.edu.cn
Xiu-Li Zuo	Department of Gastroenterology, Qilu Hospital of Shandong University	zuoxiuli@sdu.edu.cn
Hong-Wei Xu	Department of Gastroenterology, Shandong Provincial Hospital Affiliated to Shandong First Medical University	xu_hong_wei@sina.com
Qing Wang	Department of Gastroenterology, Qilu Hospital of Shandong University (Qingdao)	wangq2008001@163.com
Cheng-Xia Liu	Department of Gastroenterology, Binzhou Medical University Hospital	phdlcx@163.com
Hai-Peng Yuan	Department of Gastroenterology, Taian City Central Hospital	13375387610@163.com
Yan-Liu Chu	Department of Gastroenterology, Weihai Municipal Hospital	yanliuchu@163.com
Sen-Lin Li	Department of Gastroenterology, Liaocheng People's Hospital	lisenlin6201@163.com
Liu-Ye Huang	Department of Gastroenterology, Yantai Yuhuangding Hospital	huangliuye-yhd@163.com

Lin Lu	Department of Gastroenterology, Linyi People's Hospital	LinLu2043@163.com

3) Data and Safety Monitoring Board (DSMB)

Name	Role
Jing-Nan Li	Chair of DSMB
Ying-Xuan Chen	Voting member
Chong-Mei Yang	Voting member
Feng Chen	Voting member
Peng Chen	Voting member

2. Introduction

Colorectal cancer (CRC) is a substantial disease burden and the incidence of young-onset CRC is increasing in the United States and East Asia[1]. Screening and surveillance colonoscopy reduces the risks of CRC and related death by increasing the rates of adenoma detection and removal[2]. However, in a meta-analysis, Zhao et al. reported that up to 26% of adenomas were missed[3], and the lesions missed contributed to over 50% of the interval CRCs.

Colonoscopy efficacy to prevent CRC is widely variable due to the discrepancy of patient-, endoscopist- and procedure-related characteristics. In addition, innovation tools for increasing mucosal exposure[4–6], enhancing imaging[7,8] and wider viewing angle[9,10] are highly dependent on endoscopist observation. Therefore, different quality indicators including withdrawal time, cecal intubation, bowel preparation, photodocumentation of key landmarks, and adenoma detection rate (ADR) were

introduced to improve colonoscopy quality control[11,12]. However, these indicators are not well followed during routine colonoscopies since endoscopists' workload, insufficiency of immediate supervision and lacking practical tools. In addition, manual quality control of colonoscopy is time-consuming and subject to inter- and intra-rater variability.

Automatic quality control system (AQCS) were developed for timing of colonoscopy intubation and withdrawal phase, monitoring withdrawal stability, evaluating bowel preparation, and detecting polyps[13]. AQCS has been shown to increase the adenoma detection rate (ADR) in an academic-center randomized controlled trial of 659 participants. Several studies found significant improvements in adenoma detection with computer-aided detection devices using randomized parallel or tandem methodololgy[14,15]. Most of these tools, shown to be efficacious primarily in academic center-based trials, have not been tested whether retain their impact under non-academic settings.

1. Objective

This multicenter randomized controlled trial sought to assess the effect of the AQCS on improving the detection of colorectal adenomas in both academic and non-academic settings.

2. Outcomes

The ADR of SC and AQCS-assisted group was analyzed as the primary outcome, defined as the proportion of individuals with one or more adenomas identified by histopathology. Sessile serrated lesions (SSLs) were not considered in the ADR calculation.

Secondary outcomes included advanced ADR (AADR), proximal colonic ADR, ADR in academic and non-academic settings, adenoma per colonoscopy (APC), withdrawal

time without intervention, Boston Bowel Preparation Scale (BBPS) scores, false-positive and false-negative prompts of AQCS, adverse events, and others.

Advanced adenomas were defined as adenomas diameter ≥ 10 mm, or containing villous components, or high-grade dysplasia. The proximal colon was defined as extending from cecum to transverse colon. APC was defined as the total number of adenomas divided by the total number of colonoscopies performed in each group. Withdrawal time without intervention excluded the time spent on therapeutic interventions. The primary analysis was pre-specified to be assessed in the intention-to-treat (ITT) population, which included all randomly assigned participants. Per-protocol (PP) analysis included participants except those with incomplete colonoscopy or unqualified bowel preparation (BBPS score < 6 or subscore below 2).

5. Study design

This will be a multicenter, parallel-group, randomized controlled trial assessing the impact of AQCS on routine colonoscopy procedure. The randomization schedule was computer generated by an investigator who didn't participate in data collection or analysis, and there were no additional restrictions placed on sequence generation. Sealed opaque envelopes with computer-generated random numbers were kept by another investigator who didn't directly participate in study. All eligible patients were randomly assigned (1:1) to either standard colonoscopy or AQCS-assisted colonoscopy via block randomization stratified by center.

Intervention allocation was masked from patients, pathologists, and statistical analysts, but endoscopists were aware of group assignment.

6. Study population

The study included consecutive patients, who were scheduled to undergo routine

colonoscopy examinations in the endoscopic center of Qilu Hospital of Shandong University, Binzhou Medical University Hospital, Central Hospital of Shengli Oilfield, Zibo Municipal Hospital, Linyi People's Hospital, and the People's Hospital of Zhaoyuan City.

Inclusion criteria:

(1) patients aged between 18 and 80 years;

(2) patients undergoing colonoscopy examination.

Exclusion criteria:

(1) patients with a history of inflammatory bowel disease, advanced colorectal cancer, polyposis syndromes or known colorectal polyps without complete removal previously;

(2) patients with a history of colorectal surgery;

(3) patients with a contraindication for biopsy;

(4) patients with prior failed colonoscopy;

(5) patients with known stenosis or obstruction;

(6) patients in pregnancy or lactation phase;

(7) patients refused to participate in the trial.

Fifteen endoscopists participated in the study, with a minimum of 1500 colonoscopies before this study.

7. Study procedure

1) Trial profile

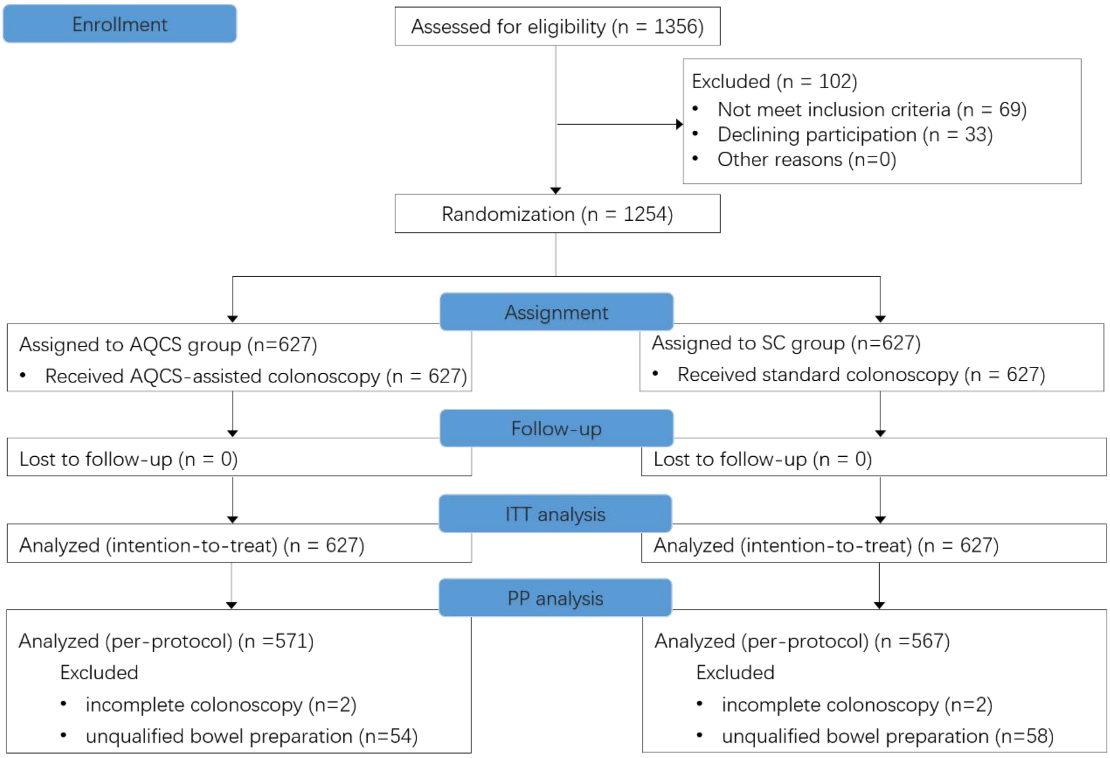

2) Procedure

All eligible patients were randomly assigned (1:1) to either standard colonoscopy or AQCS-assisted colonoscopy via block randomisation stratified by center.

High-definition endoscopy systems including the EVIS LUCERA ELITE (Olympus Medical Systems, Tokyo, Japan) and EPK-i7000(A)/EPK-i5000/EPK-3000 (Pentax Medical, Shanghai, China) were used during this study. All procedures were performed by 15 endoscopists (2-3 for each center; >1500 colonoscopies before this study) by high-definition colonoscopes (CF-HQ290, EC-3490TFi, or EC-3890Fi). Mucosal exposure devices and dye-based/electronic chromoendoscopy were not permitted for lesion detection except for characterization purposes.

Whether to undergo anesthesia during procedures is decided jointly by anesthesiologists and patients. Patient management and anesthesia monitoring were performed according to institutional protocols. Bowel preparation was recorded during

the examination, using the Boston Bowel Preparation Scale (BBPS).

The AQCS was connected with the endoscopy video processors, providing real-time audio and visual prompts via a dual monitor setup. Colonoscopy procedures were performed using the same device, with AQCS being enabled or disabled by the staff assistant according to intervention allocation. All endoscopists had received AQCS instructions from text, video and hands-on training (10 colonoscopies) before embarking on this study.

In the AQCS group, AQCS was turned on before intubation and started supervising at withdrawal phases once the cecum was identified. Alongside the original videos, four additional visual and audio notices were fed back to endoscopists in real time: (1) timer on a second high-definition monitor; (2) prompts for controlling withdrawal speed and reexamining certain segments when unsteady or fuzzy frames identified continuously by AQCS; (3) prompts for cleaning mucosa or suctioning liquid pools when suboptimal cleansing (BBPS score <2) was recognized; and (4) tracking box on the monitor indicating lesions location. The staff assistant recorded the withdrawal time without intervention using a stopwatch. Withdrawal time for negative colonoscopies (colonoscopies with no polyps, CRC, or SSL) was also calculated. Withdrawal time without intervention excluded the time spent on therapeutic interventions.

In the standard group, standard colonoscopy examinations were performed without the assistance of AQCS.

During the withdrawal phase, polyps detected with/without AQCS assistance were removed or biopsied for histopathology (except hyperplastic-appearing polyps located

in rectum with size ≤5 mm) at the discretion of the endoscopists. Patients with nonresectable lesions were recommended for therapeutic polypectomy during the study period to obtain the pathological gold standard. Polyp size was estimated with open forceps. For polyp morphology, classifications were as follows: pedunculated (Ip); semi-pedunculated (Isp); sessile or flat (Is, IIa, IIb, IIc, and III).

8. Assessment of safety

Adverse event means any untoward or unfavorable medical occurrence associated with the subject's participation in the research, whether or not considered related to the study intervention. Adverse events can be any of the following: 1) Physical signs or symptoms caused by examination or anesthesia; 2) Changes in vital signs or physical exam findings, or test results caused by examination or anesthesia; 3) An increase in the frequency or intensity (worsening) of a condition or illness that is present before study enrollment.

All observed or volunteered adverse events during the procedure or recovery, regardless of study group or suspected causal relationship to the study intervention(s), will be recorded. Sufficient information will be obtained for fully assessment of the relationship between the adverse event and the study intervention(s). Adverse events were monitored within 30 days after colonoscopy.

9. Data analysis

1) Simple size

Based on the mean ADR (24%, unpublished data) among patients undergoing colonoscopies at six involved centers over the past 12 months, a sample size of 501

subjects per group was warranted to yield an 8% increase in ADR with 80% power (5% α; two-sided test) in AQCS group. Thus, the estimated sample size was 1254 patients in total with a 20% drop-out rate.

2) Data analysis plan

All data were analyzed based on the intention-to-treat population which included all randomly assigned patients. Continuous variables are reported as mean ± SD or median with range. Numbers and percentages were used for categorical variables. Baseline clinical and colonoscopy features were compared using the chi-squared test or Fisher's exact test for the categorical variables and the two-tailed Student's *t*-test for the continuous variables. The primary and secondary outcomes were compared by mixed-effects logistic regression or negative binomial regression with endoscopist's level as the random effects, and patient's characteristics including indication for colonoscopy (screening, surveillance, and diagnosis), age, sex assigned at birth, smoking history, withdrawal time without intervention, bowel cleanliness, and basal ADR levels (<25%, 25-35%) as fixed effects. The 2-sided p value of <0·05 was judged statistically significant. No adjustment for multiple testing for exploratory endpoints was made. All data were analyzed with R v.3.6.0 or higher.

10. Ethics

This clinical trial was approved by the medical ethics committee of Qilu Hospital of Shandong University (（科）伦审第 2020 （058）号). The trial protocol was reviewed and approved by the ethical committees of all participating centers. All participated patients should provide the informed consent forms. This trial was registered with

ClinicalTrials.gov, number NCT04901130.

11. Data quality control

The independent Data and Safety Monitoring Board (DSMB) of Qilu Hospital of Shandong University will oversee the study, and review data generated from the study and revisions of the protocol prior to their implementation. Its primary objectives are to ensure the safety of study subjects and the integrity of the research data. The DSMB advises on research design issues, data quality, and research participant protections for the study.

The data manager will conduct monthly comprehensive data checks for identifying systematic errors or problems, as well as regular manual checks. Manual checks will identify more complicated and less common errors.

12. Data share

The data collected for the present study, including deidentified participant data will be made available to others. These data can be made available following communication with Zhen Li (qilulizhen@sdu.edu.cn). Before these data are shared (with professional colleagues or other investigators), the request will be reviewed by the corresponding author Zhen Li. All proposals must be approved by Zhen Li, with a signed data access agreement in hand before release of the data.

Reference

[1] Dekker E, Tanis PJ, Vleugels JLA, et al. Colorectal cancer. The Lancet 2019; 394: 1467–1480. doi:10.1016/S0140-6736(19)32319-0

[2] Robertson DJ, Lieberman DA, Winawer SJ, et al. Colorectal cancers soon after colonoscopy: a pooled multicohort analysis. Gut 2014; 63: 949–956. doi:10.1136/gutjnl-2012-303796

[3] Zhao S, Wang S, Pan P, et al. Magnitude, Risk Factors, and Factors Associated With Adenoma Miss Rate of Tandem Colonoscopy: A Systematic Review and Meta-analysis. Gastroenterology 2019; 156: 1661-1674.e11. doi:10.1053/j.gastro.2019.01.260

[4] Rex DK, Repici A, Gross SA, et al. High-definition colonoscopy versus Endocuff versus EndoRings versus full-spectrum endoscopy for adenoma detection at colonoscopy: a multicenter randomized trial. Gastrointestinal Endoscopy 2018; 88: 335-344.e2. doi:10.1016/j.gie.2018.02.043

[5] Shirin H, Shpak B, Epshtein J, et al. G-EYE colonoscopy is superior to standard colonoscopy for increasing adenoma detection rate: an international randomized controlled trial (with videos). Gastrointest Endosc 2019; 89: 545–553. doi:10.1016/j.gie.2018.09.028

[6] Facciorusso A, Del Prete V, Buccino RV, et al. Comparative Efficacy of Colonoscope Distal Attachment Devices in Increasing Rates of Adenoma Detection: A Network Meta-analysis. Clinical Gastroenterology and Hepatology 2018; 16: 1209-1219.e9. doi:10.1016/j.cgh.2017.11.007

[7] Leung WK, Lo OSH, Liu KSH, et al. Detection of Colorectal Adenoma by Narrow Band Imaging (HQ190) vs. High-Definition White Light Colonoscopy: A Randomized Controlled Trial. American Journal of Gastroenterology 2014; 109: 855–863. doi:10.1038/ajg.2014.83

[8] Repici A, Wallace MB, East JE, et al. Efficacy of Per-oral Methylene Blue Formulation for Screening Colonoscopy. Gastroenterology 2019; 156: 2198-2207.e1. doi:10.1053/j.gastro.2019.02.001

[9] Bronzwaer M, Dekker E, Weingart V, et al. Feasibility, safety, and diagnostic yield of the Extra Wide Angle View (EWAVE) colonoscope for the detection of colorectal lesions. Endoscopy 2017; s-0043-120666. doi:10.1055/s-0043-120666

[10] Papanikolaou I, Apostolopoulos P, Tziatzios G, et al. Lower adenoma miss rate with FUSE vs. conventional colonoscopy with proximal retroflexion: a randomized back-to-back trial. Endoscopy 2017; 49: 468–475. doi:10.1055/s-0042-124415

[11] Shaukat A, Kahi CJ, Burke CA, et al. ACG Clinical Guidelines: Colorectal Cancer Screening 2021. Am J Gastroenterol 2021; 116: 458–479. doi:10.14309/ajg.0000000000001122

[12] May FP, Shaukat A. State of the Science on Quality Indicators for Colonoscopy and How to Achieve Them. Am J Gastroenterol 2020; 115: 1183–1190. doi:10.14309/ajg.0000000000000622

[13] Su J-R, Li Z, Shao X-J, et al. Impact of a real-time automatic quality control system on colorectal polyp and adenoma detection: a prospective randomized controlled study (with videos). Gastrointestinal Endoscopy 2020; 91: 415-424.e4. doi:10.1016/j.gie.2019.08.026

[14] Repici A, Badalamenti M, Maselli R, et al. Efficacy of Real-Time Computer-Aided Detection of Colorectal Neoplasia in a Randomized Trial. Gastroenterology 2020; 159: 512-520.e7. doi:10.1053/j.gastro.2020.04.062

[15] Wang P, Liu P, Glissen Brown JR, et al. Lower Adenoma Miss Rate of Computer-Aided Detection-Assisted Colonoscopy vs Routine White-Light Colonoscopy in a Prospective Tandem Study. Gastroenterology 2020; 159: 1252-1261.e5. doi:10.1053/j.gastro.2020.06.023
