# Supplementary material for: Automatic Quality Control System and Adenoma Detection Rates During Routine Colonoscopy: A Randomized Clinical Trial
Source: JAMA Netw Open. 2025 Jan 30;8(1):e2457241. doi: 10.1001/jamanetworkopen.2024.57241 (PMC11783196; doi:10.1001/jamanetworkopen.2024.57241)
Supplement: Supplement 3. — Data Sharing Statement [file jamanetwopen-e2457241-s003.pdf]

Data Sharing Statement

Liu. Automatic Quality Control System and Adenoma Detection Rates During Routine Colonoscopy. *JAMA Netw Open*. Published January 30, 2025.
doi:10.1001/jamanetworkopen.2024.57241

Data
Additional Information: ClinicalTrials.gov number: NCT04901130
Data available: Yes
Data types: Deidentified participant data
How to access data: These data can be made available following communication with Zhen Li (qilulizhen@sdu.edu.cn).
When available: With publication

Supporting Documents
Document types: Other (please specify)
Additional Information: Trial protocol
How to access documents: qilulizhen@sdu.edu.cn
When available: With publication

Additional Information
Who can access the data: Researchers whose proposed use of the data has been approved.
Types of analyses: For a specified purpose.
Mechanisms of data availability: After approval of a proposal
